# A comparative evaluation of eggshell derived hydroxyapatite and demineralized freeze-dried bone allograft in alveolar ridge preservation – a randomized clinical trial

**DOI:** 10.2340/biid.v12.44941

**Published:** 2025-12-04

**Authors:** Mopati Nishanth Reddy Gokul, Konathala SV Ramesh, Penmetsa S Gautami, Naga Venkata SG Sruthima, Pasupuleti Mohan Kumar, Kanakamedala Anilkumar, Chittabathina Poornima

**Affiliations:** a Department of Periodontics, Vishnu Dental College, Bhimavaram, Andhra Pradesh, India; b Department of Periodontics and Implantology, Vishnu Dental College, Bhimavaram, Andhra Pradesh, India

**Keywords:** Alveolar ridge preservation, DFDBA, egg shell hydroxyapatite, keratinized gingiva

## Abstract

**Background:**

Alveolar ridge preservation (ARP) is critical for minimizing post-extraction bone loss and maintaining ridge dimensions essential for prosthetic replacement. Eggshell-derived hydroxyapatite (EHA), owing to its compositional similarity to natural bone and promising biological properties, has emerged as a potential alternative to conventional graft materials such as Demineralized Freeze-Dried Bone Allograft (DFDBA). The aim of the study was to compare the clinical and radiographic outcomes of EHA and DFDBA in ARP.

**Materials and methods:**

This prospective randomized clinical trial involved 20 patients requiring mandibular posterior extractions, which were assigned to two groups: EHA (test) and DFDBA (control). In both groups, bone grafts were combined with injectable Platelet-Rich Fibrin (i-PRF) to form sticky bone and sealed with Advanced Platelet-Rich Fibrin (A-PRF) membranes. Clinical parameters such as Plaque Index (PI), Gingival Index (GI), Width of Keratinized Gingiva (WKG) and radiographic parameters such as Vertical Ridge Height, Horizontal Ridge Width, and Bone Density were evaluated at baseline and Healing Index (HI) was evaluated after 2 weeks.

**Results:**

Both study groups exhibited significant improvements in PI, GI, WKG, vertical ridge height and horizontal ridge width within group with no significant difference between the groups. However, EHA demonstrated less ridge reduction compared to DFDBA with no significant changes in bone density. Wound healing at 2 weeks showed no significant difference between groups.

**Conclusions:**

EHA with platelet-rich fibrin (PRF) is an effective, affordable, and biocompatible option for ARP. EHA demonstrated greater ridge dimensional stability and similar bone density improvements compared to DFDBA, with minimal resorption and favorable healing outcomes.

## Introduction

Alveolar bone, a dynamic structure that is remodeled by osteoblasts and osteoclasts, experiences considerable resorption, that is, 30–60% in 6 months, especially during the first 3 months following tooth extraction [1]. Over a 12-month period, further remodeling may result in an average clinical and radiographic reduction of 3.87 mm and 1.21 mm, respectively, in ridge width [2]. Alveolar ridge preservation (ARP) aims to maximize bone formation in the alveoli and reduce resorption of the buccal plate and bone crest thus reducing post-extraction resorption. [1, 3].

Numerous graft materials need to be used for 4–6 months as bio-scaffolds for socket preservation [4, 5]. Considering the limitations of autogenous bone graft, allografts such as mineralized Freeze-Dried Bone Allograft (FDBA) and Demineralized Freeze-Dried Bone Allograft (DFDBA) are frequently utilized [6, 7]. The DFDBA, enriched with bone morphogenic proteins (BMPs) aids in healing and osseointegration by stimulating mesenchymal cells to differentiate into osteoblasts and by preserving space for bone regeneration [6, 8]. Outcomes are further improved when DFDBA is combined with autologous platelet concentrates (APCs), such as platelet-rich fibrin (PRF) [9]. According to histological research, DFDBA produces a greater proportion of freshly produced vital bone than FDBA [10]. The search for the ideal graft material that is economical, biocompatible, and ecologically sustainable remains ongoing in spite of these developments.

In the recent years, egg shell derived materials have gained popularity in various dental clinical applications [11–15]. Egg shell hydroxyapatite (EHA) comprises 94% calcium carbonate, organic matter, magnesium carbonate, and calcium phosphate, and is affordable and biocompatible with osteoconductive potential that promote bone growth at slow resorption rate [16]. EHA was effective in formation of new bone and increasing bone density at extracted sites. However, socket sealing preferably with biologics would guarantee successful ridge maintenance and avoid graft displacement during regeneration [14].

Autologous blood-derived products (ABPs) and bioactive substances such as APCs, enamel matrix derivative (EMD), platelet-derived growth factor (PDGF), fibroblast growth factor (FGF), and bone morphogenetic proteins (BMPs), which control inflammation and angiogenesis, are among the biologics used in bone regeneration [17]. APCs encourage angiogenesis and facilitate regeneration of periodontal intra-bony defects, furcation defects, post-extraction sockets with improved soft tissue healing either by themselves or in conjunction with graft materials [13, 18, 19]. Injectable platelet-rich fibrin (i-PRF) is gaining popularity for its versatility, used alone or with grafts to form ‘sticky bone’, a cohesive mixture where graft particles are embedded in a fibrin mesh. This enhances bone regeneration, reduces soft tissue ingrowth, and supports osteoblast recruitment, viability, and differentiation [20].

Till date EHA has received less attention compared to DFDBA in ARP, with no studies comparing EHA and DFDBA for ARP using i-PRF to form sticky bone and A-PRF as a membrane. Therefore, this study intended to methodically assess the efficacy of EHA when compared to DFDBA in combination with bioactive materials, and applied as a gel and membrane in ridge preservation.

## Materials and methods

### Study design

This study was a prospective randomized clinical trial, conducted to evaluate and compare the clinical and radiographic outcomes of eggshell derived hydroxyapatite (EDH) and DFDBA in ARP. The entire study period was from November 2023 to January 2025. All the patients were selected from those attending the outpatient pool of Vishnu dental college, Andhra Pradesh, India.

### Ethical approval

This study has been approved by the Institutional Ethics Committee, Vishnu Dental College, Bhimavaram (ref no. IECVDC/23/PG01/PI/IVV/04) and registered under the Clinical Trials Registry (ref no. CTRI/2023/08/056018). All the patients were explained about the study and a written informed consent was obtained before the inclusion. All clinical procedures were conducted in accordance with the Declaration of Helsinki and Good Clinical Practice principles.

### Sample size assessment

Sample size analysis was performed using G*Power 3.1 software based on an effect size of 0.346 with an alpha level of 0.05. Considering a 20% dropout rate, the sample size was calculated as 26 patients, and at the end of the study only 20 patients were considered for statistical analysis.

### Outcome measures

Cone beam computed tomography (CBCT) was obtained before the extraction of the tooth and 6 months after extraction to assess the radiological parameters. Bone density was assessed in Hounsfield units (HU) using CBCT (Planmecaromexis v.6.4.7 software).

#### Primary outcomes

Radiological parameters were: vertical ridge height – from the coronal most part of the crestal bone till base of the socket, horizontal ridge width – from buccal cortical plate to lingual cortical plate at the crest of alveolar bone and bone density in HU - at base or apical third of the socket.

#### Secondary outcomes

Clinical parameters were: plaque index (Silness and Loe, 1964) [21], gingival index (Loe and Silness, 1963) [21], healing index (Landry index, 1988) [22] and width of the keratinized gingiva (WKG – the distance from the gingival margin to the mucogingival junction).

### Selection criteria

Patients 18-65 years of age having a mandibular molar displaying no periapical pathology but indicated for extraction were included in the study. Smokers, pregnant or lactating women, patients with a history or present use of bisphosphonates, uncontrolled systemic diseases, and osteoarthritis individuals were excluded.

## Methodology

### Randomization and allocation

**Figure 1:**
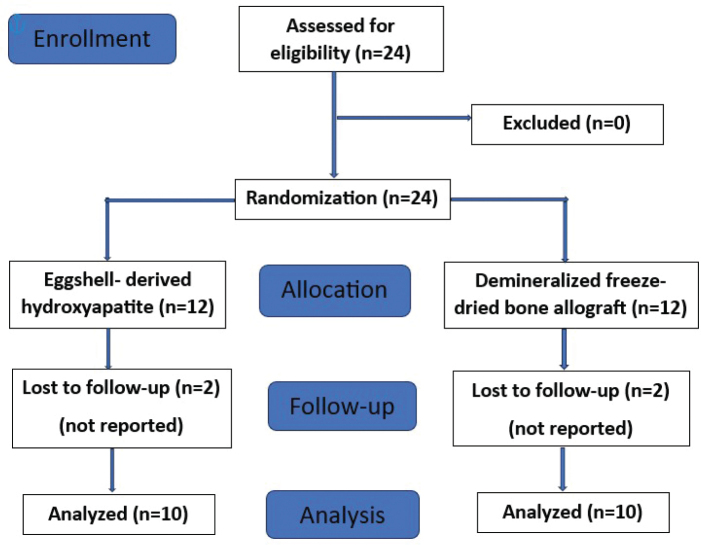
PRISMA flow chart.

All the patients underwent baseline clinical and radiographic examinations. Patient allocation was done following CONSORT guidelines (Figure 1). After fulfilling the selection criteria, randomization was done using computer generated random number tables. Twenty-four systemically healthy individuals were randomly assigned into test group (EHA) (*n* = 12) and control group (DFDBA) (*n* = 12).

### EHA preparation

EHA was indigenously prepared at Periobiologics LLP., Hyderabad. India. Initially, eggshells were processed by cleaning and removing the membranes and then washed and rinsed with distilled water, then doused with hot water for 30 min at a temperature of 100°C, and then dried for 3 days. After drying, the egg shells were crushed and mashed using a blender. A mortar was used to obtain a more delicate powder, which was then sieved using a 200-mesh sieve. Through calcination, the cleaned shells were subjected to high temperatures (between 950 and 1,000°C) for 2 h to convert calcium carbonate into calcium oxide. Hydroxyapatite synthesis was done as follows; A slurry was prepared with calcium oxide, dissolved in distilled water at 80°C for 15 min under stirring. Later, the acid solution (H_3_PO_4_) at 1M was mixed with CaO slurry and stirred at 80°C and the pH was adjusted with HCl to 9. The precipitate was washed with water, filtered and dried at 200°C for 4 h [23].

### PRF preparation

During or immediately after extraction, 20 ml (10 ml for i-PRF and 10 ml for A-PRF) of blood was drawn from the patient’s antecubital vein and collected in two sterile glass test tubes. The i-PRF tube with 10 ml of blood was placed in centrifuge at 700 rpm for 3 min, while the A-PRF tube was placed at 1,400 rpm for 13 min [20, 24].

### Pre-operative procedure

A preliminary examination, including a medical and dental history was done to evaluate each patient’s eligibility for enrolled in the study. Clinical and radiographic parameters were assessed at baseline (i.e. before extraction) and after 6 months.

### Treatment procedure

**Figure 2:**
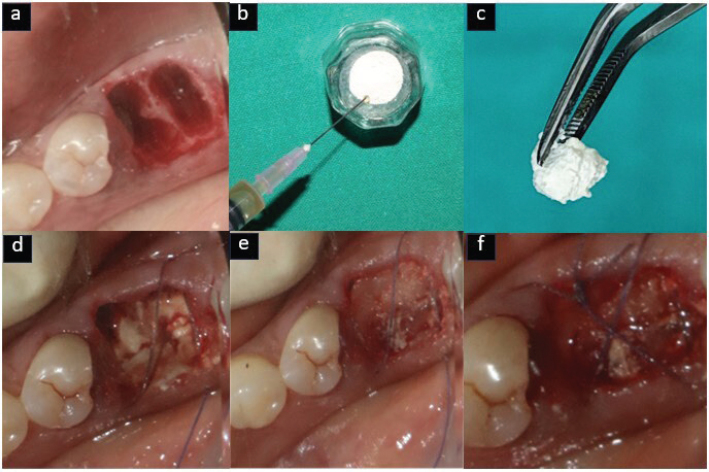
Test group (EHA) – (a) Atraumatic extraction done, (b) EHA mixed with i-PRF, (c) Sticky bone formation, (d) Sticky bone placed in socket, (e) A-PRF membrane placed, (f) sutures placed.

**Figure 3:**
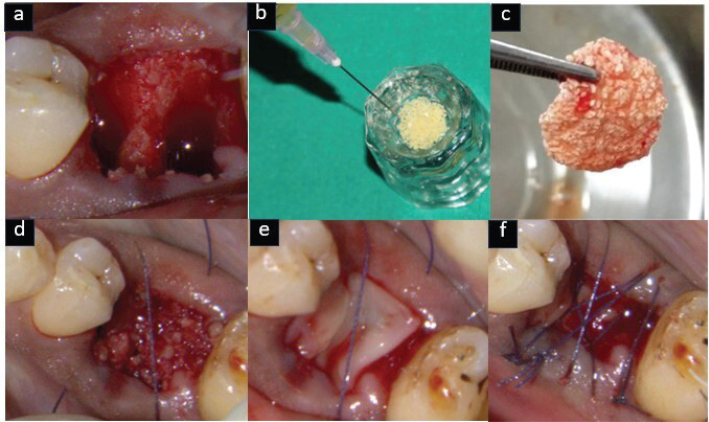
Control group (DFDBA) – (a) Atraumatic extraction done, (b) DFDBA mixed with i-PRF, (c) Sticky bone formation, (d) Sticky bone placed in socket, (e) A-PRF membrane placed, (f) sutures placed.

Atraumatic extraction was carried out under local anesthesia using 2% lignocaine hydrochloride with 1:80,000 adrenaline, employing periotomes and luxators. Socket debridement was performed with a curette followed by saline irrigation. In the test group, EHA was mixed with i-PRF and to form sticky bone, while in the control group, DFDBA was combined with i-PRF. Furthermore, the sockets were sealed with an A-PRF membrane and secured with figure-of-eight sutures using 4-0 resorbable suture material [Vicryl, Ethicon, Johnson and Johnson Ltd, India] Figures 2 and 3).

### Postoperative care

Patients were instructed to refrain from brushing at the surgical site. Antibiotics (amoxicillin 500 mg, three times daily for 3 days) and analgesics (diclofenac 50 mg, twice daily for 3 days) were prescribed. Patients were also advised to use a 0.2% chlorhexidine mouth rinse twice daily for 1 week.

### Postoperative evaluation

Following the surgical procedure, patients underwent a follow-up examination after 2 weeks for suture removal and assessment of the healing. Subsequently, patients were reevaluated after 6 moths.

### Statistical analysis

Data were analyzed using IBMSPSS, ver.26 (IBM Corp; Armonk, NY, USA). Normality of the data was assessed using Kolmogorov–Smirnov’s test. Descriptive analysis was done. Quantitative data was presented as mean ± standard deviation (SD) and qualitative data were presented as numbers and percentages. Inferential Statistics was done using independent samples t tests, paired t tests were done to analyze the study data. Data presentation was done Graph pad prisms (ver. 9.1).

## Results

Both test and control groups showed significant reductions in plaque and gingival indices from baseline to 6 months (*p* = 0.000). However, intergroup comparisons at baseline and 6 months revealed no statistically significant differences (plaque index: *p* = 0.66 and *p* = 0.46; gingival index: *p* = 0.46 and *p* = 0.40) (Table 1).

**Table 1:** Intra and Inter group comparison of plaque index, gingival index and width of keratinized gingiva at baseline, 6 months and intergroup comparison of healing index at 2 weeks.

Parameters	Study group	Mean ± SD		*P*
		Baseline	6 months	
**PLAQUE INDEX**	**Test**	1.25 ± 0.45	0.66 ± 0.31	0.000*
	**Control**	1.33 ± 0.36	0.76 ± 0.28	0.000*
	***P***	0.667	0.469	
**GINGIVAL INDEX**	**Test**	1.24 ± 0.3	0.74 ± 0.26	0.000*
	**Control**	1.36 ± 0.4	0.85 ± 0.3	0.000*
	***P***	0.463	0.401	
**WIDTH OF KERATINIZED GINGIVA (mm)**	**Test**	2.7 ± 0.67	1.6 ± 0.51	0.000*
	**Control**	2.7 ± 0.67	1.7 ± 0.38	0.000*
	***P***	1.00	0.714	
**Comparison of healing index between the study groups**				
**Parameters**	**Study group**	**Mean ± SD**	**Mean difference**	***P***
**HEALING INDEX (2 weeks)**	Test	4.4 ± 0.69		
	Control	4.1 ± 0.87	0.3	0.408

SD: standard deviation.

*p*-value < 0.05 considered statistically significant,

* denotes significance.

The width of keratinized gingiva (WKG) significantly decreased in both the test group (2.7 ± 0.67 mm to 1.6 ± 0.51 mm, *p* = 0.000) and control group (2.7 ± 0.67 mm to 1.7 ± 0.38 mm, *p* = 0.000). However, intergroup comparisons showed no significant differences at baseline (*p* = 1.00) or at 6 months (*p* = 0.71) (Table 1). At 2 weeks, the healing index was comparable between the test (4.4 ± 0.69) and control (4.1 ± 0.87) groups, with no statistically significant difference (*p* = 0.40) (Table 1).

On intragroup comparison, vertical ridge height in test group reduced from 9.78 ± 1.18 mm to 8.94 ± 1.21 mm (Δ 0.83 mm, *p* = 0.000), whereas in control group from 10.09 ± 0.84 mm to 8.49 ± 1.11 mm (Δ1.6 mm, *p* = 0.000) with a statistical significance in both the groups over 6 months. However, intergroup differences at baseline (*p* = 0.507) and 6 months (*p* = 0.398) were not statistically significant (Table 2).

**Table 2:** Intra and Inter group comparison of vertical ridge height, horizontal ridge width and bone density at baseline and 6 months.

Parameters	Study group	Mean ± SD		*P*
		Baseline	6 months	
**VERTICAL RIDGE HEIGHT (mm)**	**Test**	9.78 ± 1.18	8.94 ± 1.21	0.000*
	**Control**	10.09 ± 0.84	8.49 ± 1.11	0.000*
	***P***	0.507	0.398	
**HORIZONTAL RIDGE WIDTH (mm)**	**Test**	8.53 ± 0.97	7.65 ± 1.11	0.000*
	**Control**	8.62 ± 0.9	7.12 ± 0.98	0.000*
	***P***	0.838	0.273	
**BONE DENSITY (HU)**	**Test**	754 ± 218.8	745 ± 230.8	0.816
	**Control**	665.8 ± 149.3	681.7 ± 141.4	0.815
	***P***	0.838	0.469	

SD: standard deviation; HU: Hounsfield units.

*p*-value < 0.05 considered statistically significant,

* denotes significance.

**Figure 4:**
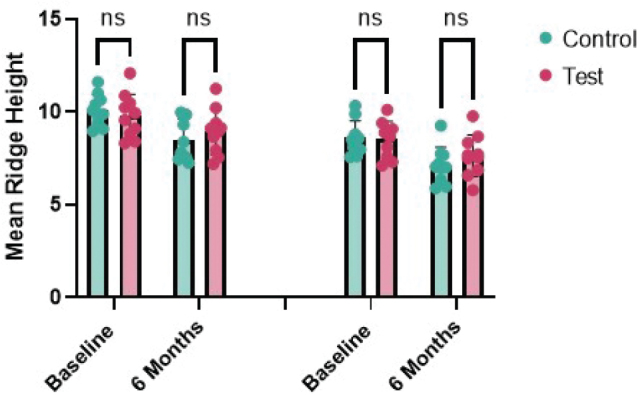
Inter-group comparison of horizontal ridge width (mm) at baseline and 6 months.

On intragroup comparison, horizontal ridge width in the test groups reduced from 8.53 ± 0.97 mm to 7.65 ± 1.11 mm (Δ 0.88 mm, *p* = 0.000) while in the control group from 8.62 ± 0.90 mm to 7.12 ± 0.98 mm (Δ 1.5 mm, *p* = 0.000) with a statistical significance in both the groups after 6 months. However, intergroup comparisons showed no significant differences at baseline (*p* = 0.838) or at 6 months (*p* = 0.273) (Table 2) (Figure 4).

**Figure 5:**
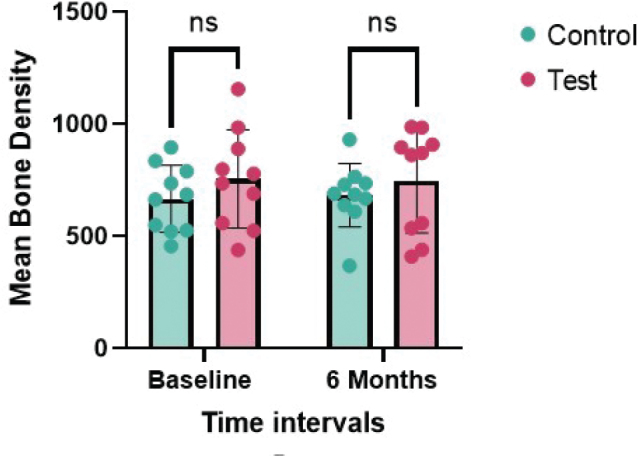
Inter-group comparison of bone density (HU) at baseline and 6 months.

**Figure 6:**
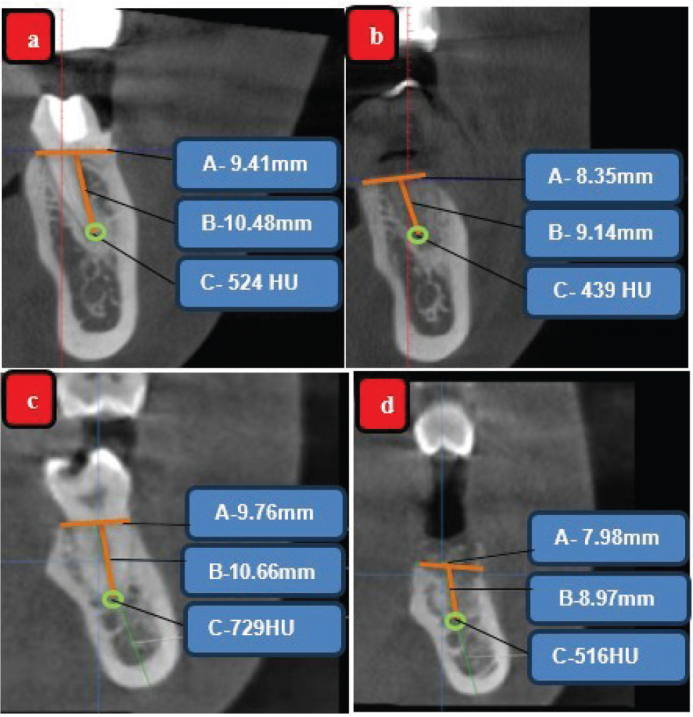
CBCT analysis (A-Horizontal bone level, B-Vertical bone level and C-bone density (HU); Test group (EHA) (a) Ridge dimensions and bone density at baseline in CBCT, (b) Ridge dimensions and bone density at 6 months in CBCT; Control group (DFDBA) (c) – Ridge dimensions and bone density at baseline in CBCT, (d) Ridge dimensions and bone density at 6 months in CBCT.

On intragroup comparison, bone density change in test group from 754.9 ± 218.8 HU to 745 ± 230.8 HU (Δ 9.9 HU, *p* = 0.816) and the control from 665.8 ± 149.3 HU to 681.7 ± 141.4 HU (Δ15.9 HU, *p* = 0.815) after 6 months were not statistically significant. Similarly, intergroup comparisons also showed no significant differences at baseline (*p* = 0.302) or at 6 months (*p* = 0.469) (Table 2) (Figure 5). The CBCT images are presented in Figure 6.

## Discussion

ARP reduces the need for invasive procedures such as grafting and ridge augmentation by preserving bone at the time of extraction, thereby supporting soft tissue contours, esthetics, and patient satisfaction [25]. Evidence indicates that combining ARP with atraumatic extraction and bone grafting effectively limits bone loss and preserves socket volume [26]. Accordingly, this study employed atraumatic extraction using periotomes to promote healing and minimize ridge resorption [27]. Common grafting materials such as DFDBA, bioactive glass, and deproteinized bovine bone are widely used in ARP but often leave residual particles long after healing [28].

EHA is an eco-friendly alternative to commercial grafts and closely mimics the composition of human bone [16, 29]. It has been effectively used in socket preservation, intra-bony defects, maxillary cysts, and apicoectomy [11–15, 29, 30]. Gutiérrez-Prieto et al. have mentioned its bioactivity and suitability for bone regeneration particularly in scaffold applications [29].

In this study, the improvement in plaque and gingival scores in both EHA and DFDBA groups indicates consistent oral hygiene practices among participants, aligning with studies reporting reduced plaque accumulation at DFDBA sites when combined with PRF [31, 32].

The WKG plays a vital role in socket grafting by minimizing plaque and inflammation to support long-term outcomes. No previous studies have assessed WKG changes with EHA in ridge preservation. In this study, both EHA and DFDBA groups showed minimal WKG reduction (1.1 mm and 1 mm, respectively), suggesting limited ridge resorption. Moghaddas et al. reported less WKG loss when DFDBA was combined with connective tissue [33]. Our study showed similar healing in both groups at 2 weeks most likely due to the consistent use of A-PRF, which promotes angiogenesis and soft tissue healing [24]. The results align with findings by Neha Nainoor and Manisha et al. [14, 32]. Notably, in this study EHA group had more cases with excellent healing (Landry score 5) than the DFDBA group.

Most alveolar bone changes occur within the first 3 months post-extraction, with horizontal resorption typically exceeding vertical loss with local and systemic factors influencing bone remodeling. Kalsi et al. reported a horizontal ridge reduction of 3.87 mm and vertical loss of 1.21 mm, both clinically and radiographically, in sockets without graft preservation [2].

In this study, atraumatic extraction was followed by socket grafting and PRF sealing to preserve the alveolar bone. EHA group showed minimal vertical ridge height reduction of 0.83 mm with 0.88 mm horizontal ridge width at 6 months. In contrast, the DFDBA group showed greater reductions of 1.6 mm vertical ridge height and 1.5 mm horizontal ridge width, aligning with Shah et al., who reported 1.38 mm vertical and 1.37 mm horizontal loss in the DFDBA group at 6 months [31].

In this study, the DFDBA group showed less horizontal ridge width compared to vertical ridge height loss, possibly due to anatomical and biological factors. Despite the rapid resorption of the thin buccal plate, ridge preservation techniques help maintain horizontal dimensions. Pooled analyses indicate that these techniques are particularly effective in minimizing horizontal bone loss [34].

In this study, baseline measurements showed that the DFDBA group had slightly greater vertical ridge height dimensions (Δ0.3 mm) and horizontal ridge width (Δ0.09 mm) compared to the EHA group. However, at 6 months, the EHA group demonstrated greater vertical ridge height (Δ0.4 mm) and greater horizontal ridge width (Δ0.5 mm) than the DFDBA group. Although the differences were not statistically significant, these results align with studies reporting greater horizontal ridge width resorption than vertical resorption [32, 35, 36] and suggest that EHA may be more effective in preserving ridge dimensions.

In this study, the EHA group showed better preservation of vertical ridge height and horizontal ridge width compared to the DFDBA group. The use of A-PRF membranes in both groups likely supported ARP. Previous studies have shown that while membranes alone may lead to greater vertical bone loss than grafts alone, they offer superior horizontal ridge preservation [34]. The best horizontal outcomes were observed when particulate grafts were combined with absorbable collagen membranes or rapidly resorbing collagen sponges [36].

Bone density plays a vital role in ARP, especially in the posterior mandible, where high masticatory forces and active remodeling occur. Despite its dense cortical nature, this region is still susceptible to trabecular resorption after extraction. While ridge height and width can be maintained, bone density varies depending on graft material and healing duration. Misch classified bone density using HU: D2 bone ranges from 850–1,250 HU and D3 from 350–850 HU, indicating differences in density and surgical implications [37, 38].

In intragroup comparison, the EHA group showed a mean decrease of 9.9 HU, while the DFDBA group exhibited a mean gain of 15.9 HU at 6 months. Similar studies have reported increased bone density with DFDBA, likely due to its osteoconductive properties and the presence of bone morphogenetic proteins that enhance osteoinduction [9, 39]. Other research also supports the effectiveness of eggshell-derived nano-hydroxyapatite (EnHA) in improving bone density in periodontal defects and socket preservation [12–15, 40].

Intergroup comparison showed no statistically significant difference in bone density, but radiologically, EHA demonstrated a slight advantage over DFDBA with a mean increase of 63.3 HU at 6 months. This aligns with findings by Neha Nainoor and Kattimani et al., who reported improved bone density with EHA over DFDBA and placebo grafts [12–14]. Conversely, Khan et al. observed greater bone density gains in the DFDBA + PRF group compared to natural healing [9].

The superior ridge preservation seen with EHA over DFDBA is due to EHA’s biological stability, slower resorption, and high osteo-conductivity. Its composition closely resembles natural bone, providing a durable scaffold that supports sustained bone formation [12–14]. The porous microstructure enhances space maintenance, cell infiltration, and vascularization, aiding both horizontal and vertical ridge preservation. Its high calcium content and gradual ion release stimulate osteoblastic activity and mineralization, promoting increased bone density [41].

A key strength of this study is its novel use of i-PRF with EHA to form sticky bone for ridge preservation. It is also the first to assess both horizontal and vertical ridge dimensions, along with keratinized gingival width, using EHA.

## Conclusions

EHA with PRF is an effective, affordable, and biocompatible option for ARP. EHA demonstrated greater ridge dimensional stability and similar bone density improvements compared to DFDBA, with minimal resorption and favorable healing outcomes. The combination of EHA osteoconductive scaffold and PRF regenerative potential supports enhanced bone formation and soft tissue healing. While the results are encouraging, future studies with larger sample sizes, longer follow-up periods, and histological analysis are needed to confirm these findings and establish long-term clinical efficacy.

Key limitations of this study include a small sample size and short 6-month follow-up, limiting statistical power and long-term insights. Furthermore, the absence of histological analysis prevents assessment of bone quality and using A-PRF as a membrane in both groups could have obscured differences between graft materials.

## Declaration of interest

The authors do not have any financial interests, either directly or indirectly, in the products or information listed in the article.

## Authors’ contributions

Conceptualization – MNRG, KSVR; Data curation-MNRG, KSVR, NVSG, PMK; Formal analysis – MNRG, KSVR; Investigation-MNRG, KSVR, ChP; Methodology – MNRG, KSVR, PSG; Project administration – MNRG; Software-MNRG, KSVR; Validation – KSVR, PSG, NVSG, PMK; Writing original draft – MNRG, KSVR; Writing – review & editing – MNRG, KSVR, NVSG, KAK.

## Data availability statement

All the data related to the current research are available in the manuscript. No additional information is required to be available in other sources.
